# Proteomic Response of *Bacillus subtilis* Spores under High Pressure Combined with Moderate Temperature and Random Peptide Mixture LK Treatment

**DOI:** 10.3390/foods11081123

**Published:** 2022-04-13

**Authors:** Yaru Pang, Ruobin Wu, Tianlin Cui, Zequn Zhang, Li Dong, Fang Chen, Xiaosong Hu

**Affiliations:** 1 College of Food Science and Nutritional Engineering, China Agricultural University, Beijing 100083, China; terpsichorean75@163.com (Y.P.); wuruobin21@outlook.com (R.W.); tianlincui@163.com (T.C.); zqzhang1995@126.com (Z.Z.); li_dong127@163.com (L.D.); chenfangch@sina.com (F.C.); 2 China National Engineering Research Center for Fruit and Vegetable Processing, Beijing 100083, China; 3 Key Laboratory of Fruits and Vegetables Processing, Ministry of Agriculture, Beijing 100083, China; 4 Engineering Research Centre for Fruits and Vegetables Processing, Ministry of Education, Beijing 100083, China

**Keywords:** *Bacillus subtilis* spores, high-pressure processing, random antimicrobial peptide mixtures, food sterilization, spore inactivation, proteomic

## Abstract

In this study, a method of *Bacillus subtilis* spore inactivation under high pressure (P, 200 MPa) combined with moderate temperature (T, 80 °C) and the addition of antimicrobial peptide LK (10^2^ μg/mL) was investigated. Spores presented cortex hydrolysis and inner membrane (IM) damage with an 8.16 log reduction in response to treatment with PT-LK, as observed by phase-contrast and inverted fluorescence microscopy and flow cytometry (FCM) analysis. Furthermore, a tandem mass tag (TMT) quantitative proteomics approach was utilized because Liquid chromatography-tandem mass spectrometry (LC–MS/MS) analysis data were used. After treatment with PT-LK, 17,017 polypeptides and 3166 proteins were detected from *B. subtilis* spores. Among them, 78 proteins showed significant differences in abundance between the PT-LK-treated and control groups, with 49 proteins being upregulated and 29 proteins being downregulated in the PT-LK-treated group. Genetic information processing, metabolism, cellular process, and environmental information processing were the main mechanisms of PT-LK-mediated spore inactivation.

## 1. Introduction

Since Nicolas-François Appert invented the first food sterilization device, food sterilization technology has undergone extensive development. The traditional thermal sterilization process with a high temperature and long operation time may lead to loss of sensorial quality and nutrients and derive undesirable compounds via rise hyperthermia reactions such as acrylamide (AA), furan and its methyl derivatives, 5-hydroxymethyl-2-furfural (HMF), glycidyl esters, and 3-MCPD esters [1]

In the past 30 years, high-pressure processing (HPP) technology has been increasingly used as a nonthermal processing method in the food industry to provide minimally processed, more nutritious, healthy, safe, fresh-tasting, and shelf-stable products. However, HPP alone still has limitations toward some bacterial spores that are highly resistant to pressure [2]. Researchers are, therefore, working to find an effective way to kill spores during HPP treatment.

Today, a better approach is combining multiple technologies to induce spore germination and then killing them. Pressure combined with other treatments creates a synergistical effect on the inactivation of spores. From 0.1–700 MPa and from low to high temperatures, scientists have extensively studied the effects of temperature and pressure synergies [3]. Early examples of research on pressure-assisted thermal processing (PATP) established that *Geobacillus stearothermophilus* spore inactivation is 4 log at 600 MPa combined with 90 °C within 6–8 min in different food systems, and *Bacillus amyloliquefaciens* spore inactivation is 5 log in different food systems for 600 MPa combined with 110 °C within 2–3 min [4]. An inactivation of 3–4 log under high pressure (300–400 MPa) combined with moderate temperature and a pressure dwell time ≥40 min indicated an optimum condition of spore germination. Combined with flow cytometric (FCM) analysis, researchers assume that spores treated under lower pressure might proceed through stage II of germination, since a fully hydrated inner membrane (IM) shows greater sensitivity to pressure. Protein synthesis and de novo RNA synthesis are disabled, which could induce misfolded proteins or lethal defects in the RNA sequence [5]. In addition, pressure combined with moderate heat and some antimicrobial compounds such as nisin and lysozyme could the enhance synergistic inactivation of *Bacillus* spp. and *Clostridium* spp. spores [6,7,8,9,10]. In previous studies, the inactivation of *B. sporothermodurans* spores was achieved using the combined treatment of 400 MPa and 121 UI·mL^−1^ nisin at a moderate temperature of 53 °C for 5 min, with 121 UI·mL^−1^ nisin resulting in a 5 log reduction [9]. Roberts [7] reported that more than 6 log spores in buffer at pH 4.0 were inactivated after the treatment of 400 MPa for 30 min in a pH 4.0 buffer at 70 °C with 0.8 UI·mL^−1^ nisin contained in nutrient agar. A study by Hofstetter [8] proved that membrane-active antimicrobial nisin accelerates dipicolinic acid (DPA) release via pores, which in turn facilitates spore inactivation under high pressure combined with high temperature.

Host defense peptides (HDPs) or natural antimicrobial peptides (AMPs) are formed from the innate immune systems of eukaryotes and operate as a defensive weapon against bacterial infection [11,12]. By disrupting bacterial cell membranes, causing them to undergo phase changes and form pores, the peptide is transported to the interior of the bacteria [12]. AMPs display more sensitive prokaryotic membranes than eukaryotic membranes [13]. Since the outer surface of prokaryotic cells usually has a greater net negative charge than that of eukaryotic cells, this selectivity is thought to be due to the net cationic charge shared by AMPs [14]. Large amounts of hydrophobic residue in AMPs might mediate disruptive interactions within the hydrophobic interior of the lipid bilayer [13].

More and more AMPs have been reported to replace nitrite as an effective safe preservative in the food industry in recent years [15,16,17]. Some peptides and bacteriocins possess high antimicrobial activity at very low concentrations toward Gram-negative and Gram-positive bacteria, including spores [18,19], and even fungi and viruses [11], mainly due to their damage to microbial cell membranes. Accordingly, random antimicrobial peptide mixtures (RPMs), which are made of hydrophobic and cationic amino acids randomly mixed in a defined proportion, were introduced. There have been several investigations into the antimicrobial activities of LK (a kind of RPM that mixes l-leucine and l-lysine as the hydrophobic residue and cationic residue, respectively) toward both Gram-negative and Gram-positive bacteria [11,20], including some spore-forming bacteria. It has been shown that LK is effective against *B*. *subtilis*, *L. monocytogenes*, *P. putida*, and *E. coli*, and it has great potential for use in meat production [11]. The minimum inhibitory concentration (MIC) values for *Bacillus subtilis* have been measured as 3 μg/mL [20] and 13 μg/mL [11] in minced turkey meat. However, there is no report about the effect of HHP combined with AMPs on spore inactivation.

Moderate pressure (200–500 MPa) can induce more spore germination than high pressure (500–600 MPa), and germinated spores can be inactivated by a temperature of 80 °C. Thus, in this study, for the first time, we treated *B. subtilis* spores with a moderate high-pressure thermal (PT) atmosphere and LK to establish a synergistical effect on spore inactivation. Furthermore, the structure change was further studied with microscopy and flow cytometry (FCM) analysis. Lastly, we utilized a tandem mass tag (TMT) quantitative proteomic analysis technique to investigate the cellular proteome profiling of *B. subtilis* spore in response to PT-LK treatment, thus elucidating the mechanism of spore inactivation by PT-LK at the protein level.

## 2. Materials and Methods

### 2.1. B. subtilis Strain and Sporulation

*B. subtilis* 168 (CGMCC 1.1087, China General Microbiological Culture Collection Center, Beijing, China) strains were cultured in LB (Luria Bertani, Difco LB Broth, Lennox, Becton, Dickinson and Company, Sparks, MD, USA) broth medium until an optical density at 600 nm (OD_600_) of 1–2. Spores were grown for around 2–3 days at 37 °C on 2 × SG (double-strength Schaeffer’s glucose) medium agar plates and collected using sterile water once more than 95% of the spores were released from vegetative cells [21]. Then, these spores were rinsed three times with sterile water and purified using the Histodenz (Alere Technologies AS, Oslo, Norway) gradient centrifugation method. All spores were kept in the dark at 4 °C or frozen at −20 °C or −80 °C [22].

### 2.2. HPP Equipment and Treatment

**Figure 1:**
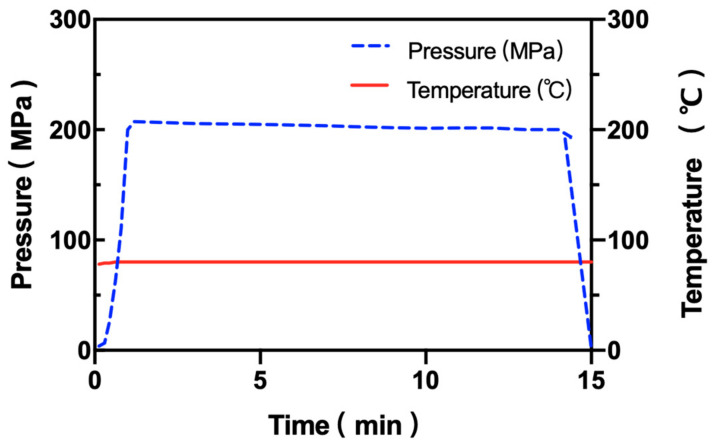
Pressure and sample temperature for a 200 MPa, 80 °C treatment with a 15 min pressure dwell time.

A high-pressure assisted thermal device (HPP 600/5 L, Bao Tou KeFa High Pressure Technology Co., Ltd., Baotou, China) (Figure 1) with a compression rate of 3.37 MPa/s and a vessel volume of 5 L by water served as the pressure transfer medium. The maximum pressure was 600 MPa. The pressure cavity was surrounded by a heater set with an upper limit temperature of 100 °C.

The high-pressure vessel was preheated to 80 °C, and the working pressure was set to 200 MPa for 15 min. In each pressure-assisted heat treatment study, sterilized polyamide/polyethylene plastic bags (4 × 10 cm) were filled with 2 mL of spore suspension. No leakage or sample loss was observed in this research.

### 2.3. RPM Synthesis

The RPM LK, a combination of hydrophobic and cationic residues including l-leucine (L) as the hydrophobic residue and l-lysine (K) as the cationic amino acid, was synthesized according to Palman and Hayouka’s [11,20] method by GL Biochem (GL Biochem Ltd., Shanghai, China) using the fmoc solid-phase peptide synthesis (SPPS) method. Nitrogen was blow-dried on rink amide resin (substitution 0.53 mmol/g, 25 μmol). Coupling reactions with binary mixes of protected amino acids with freshly prepared stock solutions containing protected amino acids in a 1:1 molar ratio of each amino acid, i.e., l-leucine and l-lysine (25 μmol), were used in each coupling step. After synthesis was completed (20 cycles, 20 peptide chain length), LK was cleaved from the resin in DMF (*N*,*N*-dimethylformamide, Sigma-Aldrich, St. Louis, MO, USA) and purified using column C18 (10 × 250 mm) and Reversed-phasehigh-performanceliquidchromatography (RP-HPLC) chromatography. Then, LK was freeze-dried. The purity of the peptide was 95.83%. Various concentrations of LK were dissolved with sterile water and stored in brown sterile centrifuge tubes at 4 °C.

### 2.4. Spore Enumeration

The colony counting method was used to determine the number of surviving spores, which were serially diluted with sterile water using a pour-plate on nutrient agar and enumerated in duplicate. All plates were incubated at 37 °C for 18–24 h until there was no increase in the number of colonies. Untreated spores were used as control conditions for the experiment to acquire the initial spore counts. The spore survival log amount [log10 (N_0_/N_t_)] was taken after treatment, where N_0_ is the initial spore count, and N_t_ is the spore count after treatment [23].

### 2.5. Microscope Analysis

The spore suspensions at an OD_600_ of 1 were double-stained with 15 µM propidium iodide (PI) (Biotopped, Beijing, China). A membrane dye was used as a break indicator for rupture of the IM and spore inactivation. Maximal values for the absorption and fluorescence emissions of the complex with nucleic acids were observed at 535 and 617 nm, respectively, using 0.5 µM SYTO16 (Invitrogen, Carlsbad, CA, USA). A membrane permeant can indicate germination when a spore’s cortex degradation occurs [23]. Maximal values for the absorption and fluorescence emissions of the complex with DNA were observed at 488 and 518 nm, respectively, and mixed vigorously [24] before incubating in the dark for 15 min prior to analysis. The spore suspensions were centrifuged (12,000× *g*, 3 min, 4 °C) after double-dyeing and observed by phase-contrast and inverted fluorescence microscopy (Axio Observer. A1, Carl Zeiss, Oberkochen, Germany) equipped with an oil immersion objective (100×/1.25 Oil Ph3 M27, N-Achroplan, NY, USA).

### 2.6. Flow Cytometry (FCM) Analysis

FCM analysis was performed using an FACS Calibur™ Flow Cytometer (BD Biosciences, San Jose, CA, USA) fitted with a 15 mW air-cooled 488 nm argon ion laser after treatment. BD CellQuest Pro (BD Biosciences) was the operating software. Sample acquisition was set to 30,000 events at a flow rate of 1000 events/s. Spore suspensions were diluted to a final concentration of approximately 10^6^ CFU/mL, and the double-dyeing operation was the same as for microscopy analysis. PI staining with red fluorescence and SYTO 16 staining with green emission were collected through a 585 and 530 nm bandpass filter, respectively [5].

### 2.7. Protein Extraction, Digestion, and TMT Quantitation

The spore suspensions at an OD_600_ of 20 were lysed, and their protein was extracted with an SDT (4% SDS, 1 mM DTT, 100 mM Tris-HCl, pH 7.6) buffer. A BCA Protein Assay Kit (Bio-Rad, Hercules, CA, USA) was used to quantify the amount of protein. Protein digestion by trypsin was performed according to the filter-aided sample preparation (FASP) procedure. The digested peptides of each sample were desalted on C18 Cartridges (SPE Cartridges C18 (standard density), bed I.D. 7 mm, volume 3 mL, Sigma-Aldrich, St. Louis, MO, USA), concentrated by vacuum centrifugation, and reconstituted in 40 µL of 0.1% formic acid.

About 200 μg of protein from each sample was incorporated into 30 μL of SDT buffer (4% SDS, 100 mM DTT, 150 mM Tris-HCl pH 8.0). Detergents, DTT, and other low-molecular-weight components were removed by repeated ultrafiltration (Microcon unit, 10 kD) using UA buffer (8 M urea, 150 mM Tris-HCl, pH 8.0). Then, 100 μL of iodoacetamide (100 mM IAA in UA buffer) was added to block the reduced cysteine residues, and the samples were incubated in the dark for 30 min, before washing three times with 100 μL of UA buffer, and then twice with 100 μL of 25 mM NH_4_HCO_3_ buffer. Finally, the protein suspension was digested with 4 μg of trypsin (Promega, Beijing, China) in 40 μL of 25 mM NH_4_HCO_3_ buffer overnight at 37 °C, and the resulting peptides were collected as filtrate. Peptides from each sample were desalted in C18 cartridges (SPE Cartridge C18 (standard density), 7 mm bed inner diameter, 3 mL volume, Sigma-Aldrich, St. Louis, MO, USA), concentrated by vacuum centrifugation, and concentrated in 40 μL of 0.1% (*v*/*v*) reconstituted formic acid. Peptide content was estimated by UV spectral density at 280 nm, using an extinction coefficient of 1.1 for a 0.1% (g/L) solution calculated from the frequencies of tryptophan and tyrosine in vertebrate proteins. About 100 μg of the peptide mixture per sample was labeled with isobaric tags for relative and absolute quantification (iTRAQ) reagent according to the manufacturer’s instructions (Applied Biosystems, Thermo Fisher Scientific, Waltham, MA, USA).

### 2.8. Peptide Fractionation

Labeled peptides were fractionated using the High-pH Reverse-Phase Peptide Fractionation Kit (Thermo Fisher Scientific, Waltham, MA, USA). Peptide powders were reconstituted, acidified with 0.1% TFA solution, and then loaded into an equilibrated high-pH reverse-phase fractionation spin column. The peptide was bound to a hydrophobic resin under aqueous conditions and desalted by washing the column with water under low-speed centrifugation. Then, a gradient of acetonitrile concentration was added to the volatile high-pH eluent, and the bound polypeptide was eluted into 10 different fractions, which were collected by centrifugation. The collected fractions were desalted on a C18 column (Empore™ SPE column C18 (standard density), 7 mm bed diameter, 3 mL volume, Sigma-Aldrich, St. Louis, MO, USA) and concentrated by vacuum centrifugation.

### 2.9. LC–MS/MS Analysis

LC–MS/MS analysis was performed on a Q Exactive Mass Spectrometer (Thermo Scientific) coupled to Easy nLC (Proxeon Biosystems, now Thermo Fisher Scientific, Waltham, MA, USA)) for 60/90 min. The peptides were loaded onto a reverse-phase trap column (Thermo Scientific Acclaim PepMap100, 100 μm × 2 cm, nanoViper C18), which was connected to a C18 reverse-phase analytical column (Thermo Scientific Easy Column, 10 cm long, 75 μm inner diameter, 3 μm resin), separated by a linear gradient of buffer B (84% acetonitrile and 0.1% formic acid) in buffer A (0.1% formic acid) at a flow rate of 300 nL/min controlled by IntelliFlow technology. The mass spectrometer was run in positive ion mode. MS data were acquired using a data-dependent top 10 method to dynamically determine the most abundant precursor ions from the survey scan (300–1800 *m*/*z*) for HCD fragmentation. The automatic gain control (AGC) target was set to 3 × 10^6^, and the maximum inject time was set to 10 ms. The dynamic exclusion duration was 40.0 s. The resolution of the survey scan was 70,000 at 200 *m*/*z*, and the resolution for HCD spectra was set to 17,500 at 200 *m*/*z*, with the isolation width of 2 *m*/*z*. The normalized collision energy was 30 eV, and the underfill ratio, which specifies the minimum percentage of the target value likely to be reached at maximum fill time, was defined as 0.1%. The instrument was run with peptide recognition mode enabled.

### 2.10. Data Analysis

All experiments had three biological replicates, including those for the control and HPP-treated groups, and data were presented as the mean ± standard deviation. One-way analysis of variance (ANOVA) was used to test the statistically significant differences (*p* < 0.05) between treatments using SPSS (SPSS Statistical for Mac, version 26.0.0.2, IBM, Armonk, NY, USA). Plots and a fitted mathematical model were created using GraphPad Prism (GraphPad Prism 9 for Mac, version 9.3.1, GraphPad Software, San Diego, CA, USA). For TMT quantitation analysis, all raw data of MS for each sample were searched using the MASCOT engine (Version 2.2, Matrix Science, London, UK) embedded into Proteome Discoverer software (Version 1.4, Thermo Fisher Scientific, Waltham, MA, USA) for identification and quantitation analysis. The protein sequence was obtained from Uniprot. The InterProScan (Version-5.25-64.0, Honeywell International, NJ, USA) software was used to search protein sequences to identify protein domain signatures from the InterPro member database Pfam. NCBI BLAST+ was used to locally search the protein sequences of the selected differentially expressed proteins, and InterProScan was used to find homolog sequences, after which gene ontology (GO) terms were mapped, and sequences were annotated using the software program Blast2GO (Version2.2.25+, NCBI, USA). Following annotation steps, all studied proteins were blasted against the Kyoto Encyclopedia of Genes and Genomes (KEGG) database (https://www.kegg.jp/ (6 January 2022)) to retrieve their KEGG Orthology identifications, and they were subsequently mapped to pathways in the KEGG.

Enrichment analysis was applied on the basis of Fisher’s exact test considering the whole quantified proteins as the background dataset. Benjamini–Hochberg correction for multiple testing was then applied to adjust derived *p*-values. Only functional categories and pathways with *p*-values under 0.05 were considered significant.

## 3. Results

### 3.1. Inactivation Effect of PT-LK on B. subtilis Spores

**Figure 2:**
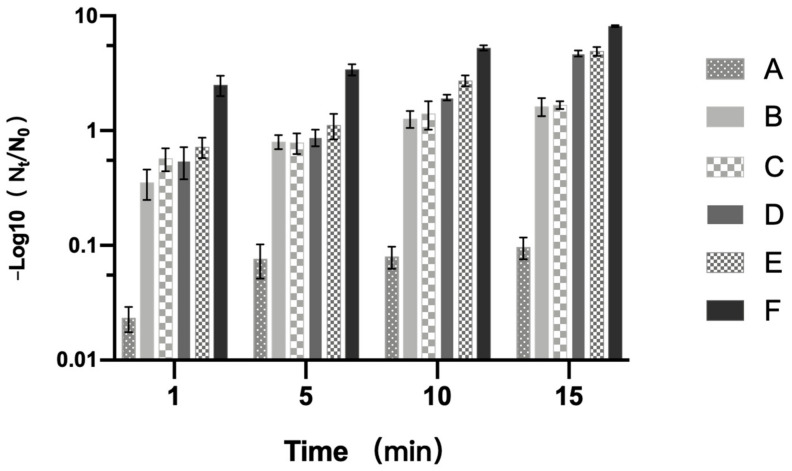
Effects of different concentrations of LK combined with PT on spore inactivation. (**A**) Untreated; (**B**–**F**) treated at 200 MPa and 80 °C combined with LK at concentrations of 0, 10^−2^, 1, 10, and 10^2^ μg/mL, respectively.

To explore the effects of treatment at 200 MPa and 80 °C combined with 0, 10^−2^, 1, 10, and 10^2^ μg/mL LK within 15 min on the inactivation of *B. subtilis* spores, the plate counting experiment was implemented. The results (Figure 2) showed that PT-LK treatment with a low LK level of 10^−2^ μg/mL for 1, 5, 10, and 15 min achieved 0.57 log, 0.79 log, 1.41 log, and 1.68 log spore reduction, respectively. When the LK concentration was increased to 10^2^ μg/mL, the PT-LK treatment achieved spore reductions of 2.5 log, 3.41 log, 5.26 log, and 8.16 log for 1 min, 5 min, 10 min, and 15 min, respectively. These results indicated that the spore inactivation rate increased with the extension of treatment time, and the inactivation effect was the best with the PT-LK treatment for 15 min. In addition, the spore inactivation was 1.63 log, 1.68 log, 4.72 log, 4.93 log, and 8.16 log with 0, 10^−2^, 1, 10, and 10^2^ μg/mL LK combined with the high-pressure treatment of 200 MPa at 80 °C for 15 min. These results showed that the spore inactivation varied from 1.68 to 8.16 log with the increase in concentration of LK from 10^−2^ to 10^2^ μg/mL. All of these findings suggested that spore inactivation depended on the treatment time and the concentration of LK.

### 3.2. Morphological Changes in Spores in Response to PT-LK

**Figure 3:**
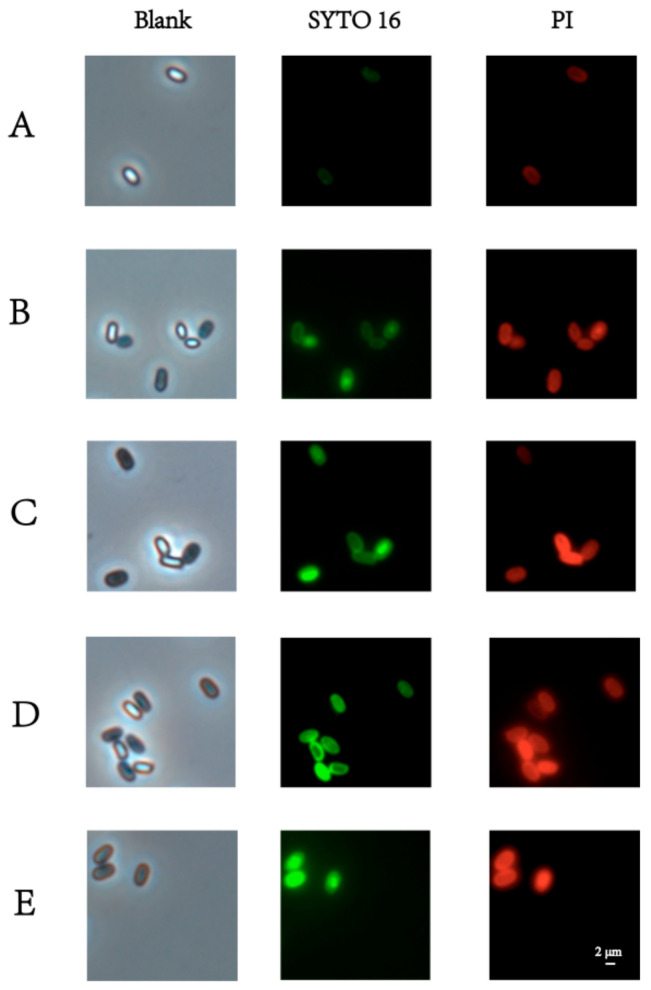
Phase-contrast and fluorescence microscope images of (**A**) untreated spores and spores treated with (**B**) 200 MPa for 15 min, (**C**) 80 °C for 15 min, (**D**) LK at 25 °C for 15 min, and (**E**) 200 MPa at 80 °C combined with LK for 15 min. The concentration of LK was 10^2^ μg/mL.

The physiological states of the PT-LK-treated spores were identified under the phase-contrast microscope (Figure 3). Compared with untreated spores, it can be found that only some spores changed their brightness from bright to gray after being individually treated by pressure, thermal, or LK, whereas all turned gray after PT-LK treatment. This means that only a small proportion of spores were hydrated [25] by individual pressure, thermal, or LK treatment, whereas they were all germinated by PT-LK treatment. Green fluorescence from SYTO 16 staining and red fluorescence from PI staining are often used as indicators of cortex hydrolysis and damage to the IM, respectively [26]. Compared to untreated spores, only those treated with pressure, heat, or LK (Figure 3A–C) were partly stained by PI and SYTO 16, signifying that the spore IMs and cortexes were partially damaged. Interestingly, all spores that were stained by both PI and SYTO 16 can be observed in Figure 3E, indicating that the spore IMs and cortexes were all damaged under PT-LK treatment.

**Figure 4:**
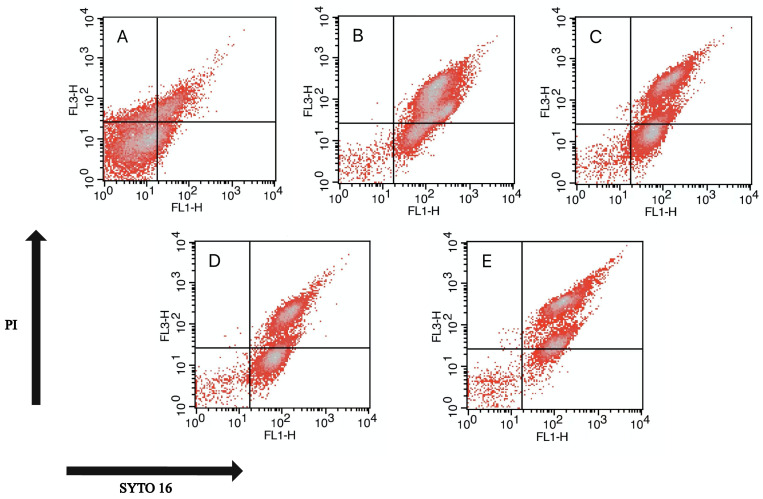
Flow cytometer density plot diagrams of (**A**) untreated spores and spores treated with (**B**) 200 MPa for 15 min, (**C**) 80 °C for 15 min, (**D**) LK at 25 °C for 15 min, and (**E**) 200 MPa at 80 °C combined with LK for 15 min. The concentration of LK was 10^2^ μg/mL.

To compare the staining characteristics of spore samples, flow cytometry analysis was used to detect the different fluorescence intensities (Figure 4), and the percentages of spores in different states are shown in Table 1. Spores in the untreated group with intact cortex and IM structures were almost unstained and concentrated in the LL (lower left) region (Figure 4A), where the subpopulation was assigned as the dormant (culturable) spore [26]. Both pressure and thermal treatment groups (Figure 4B,C) revealed higher-intensity SYTO 16 staining than PI staining, mainly concentrated in the UR (upper right) and LR (lower right) regions, as the subpopulation in UR was assigned as the germinated (culturable, but heat-sensitive) spores, whereas that in the LR was assigned as the unknown subpopulation. The results indicated that the IMs still maintained very low permeability. These results are similar to those reported by Zhang [27]. However, the spore cortex injuries were comparable to the IM injuries when treated with LK only (Figure 4D). PT-LK treatment with the highest intensity for SYTO 16 staining and PI staining (Figure 4E) was mainly concentrated in the UR region, where damage to both the spore cortex and the IM changed significantly.

### 3.3. TMT Proteomic Analysis of B. subtilis Spores in Response to PT-LK

**Figure 5:**
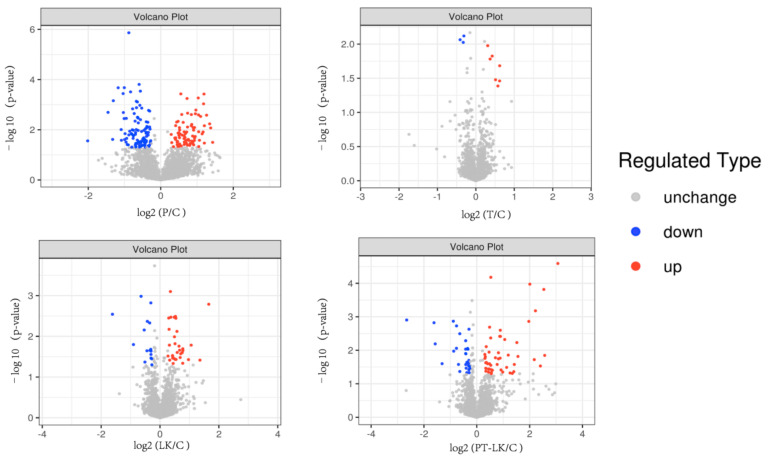
Volcano plot of changes in the levels of identified *B. subtilis* spore proteins analyzed using TMT quantitative proteomics after different treatments.

**Figure 6:**
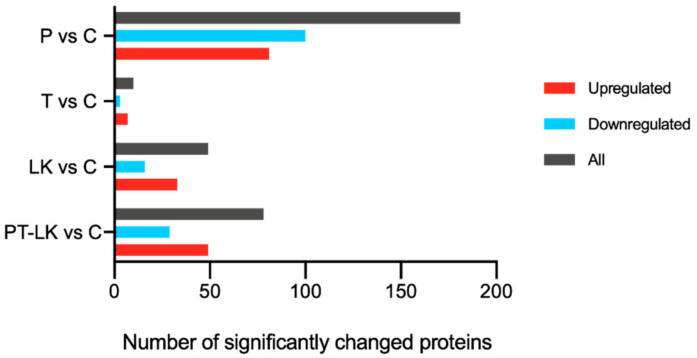
Proteomic changes in *B. subtilis* spores in response to different treatments.

In view of *B. subtilis* having nearly 75% spore dry weight content, proteins play a very important role in spore resistance [28]. It is, therefore, meaningful to identify and characterize spore proteins in response to the synergistic effects of PT-LK treatment. Proteomic analysis in this research was conducted using a TMT quantitation approach. In total, 17,017 peptides and 3116 proteins were detected from *B. subtilis* spores given PT-LK treatment, and proteins with a fold change (FC) > 1.2 and *p* < 0.05 were considered to have significantly changed in abundance. Proteins with different levels in pressure (P) under 200 MPa for 15 min/control (C), temperature (T) at 80 °C for 15 min/C, 10^2^ μg/mL LK (LK) at 25 °C for 15 min/C, and PT-LK/C comparisons are illustrated in Figure 5. As can be seen from Figure 6, pairwise comparison of spore protein levels among P/C, T/C, LK/C, and PT-LK/C identified 181, 10, 49, and 78 differentially expressed proteins (DEPs), respectively. There were 81, seven, 33, and 49 proteins upregulated among these groups, whereas 100, three, 16, and 29 proteins, respectively, were downregulated.

### 3.4. Gene Ontology (GO) Analysis of B. subtilis Spore Proteins Induced by PT-LK

For the facilitation of a comprehensive understanding of the function, localization, and involved biological pathways of proteins in living organisms, the proteins were annotated by GO, a standardized functional classification system that provides a dynamically updated set of standardized vocabulary to describe the properties of genes and gene products in an organism. The GO functional annotation mainly includes biological processes (BP), molecular functions (MF), and cellular components (CC) [29].

**Figure 7:**
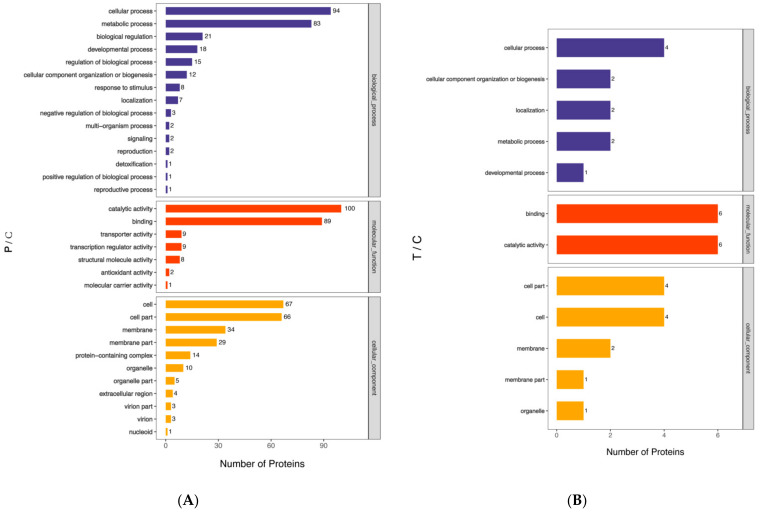

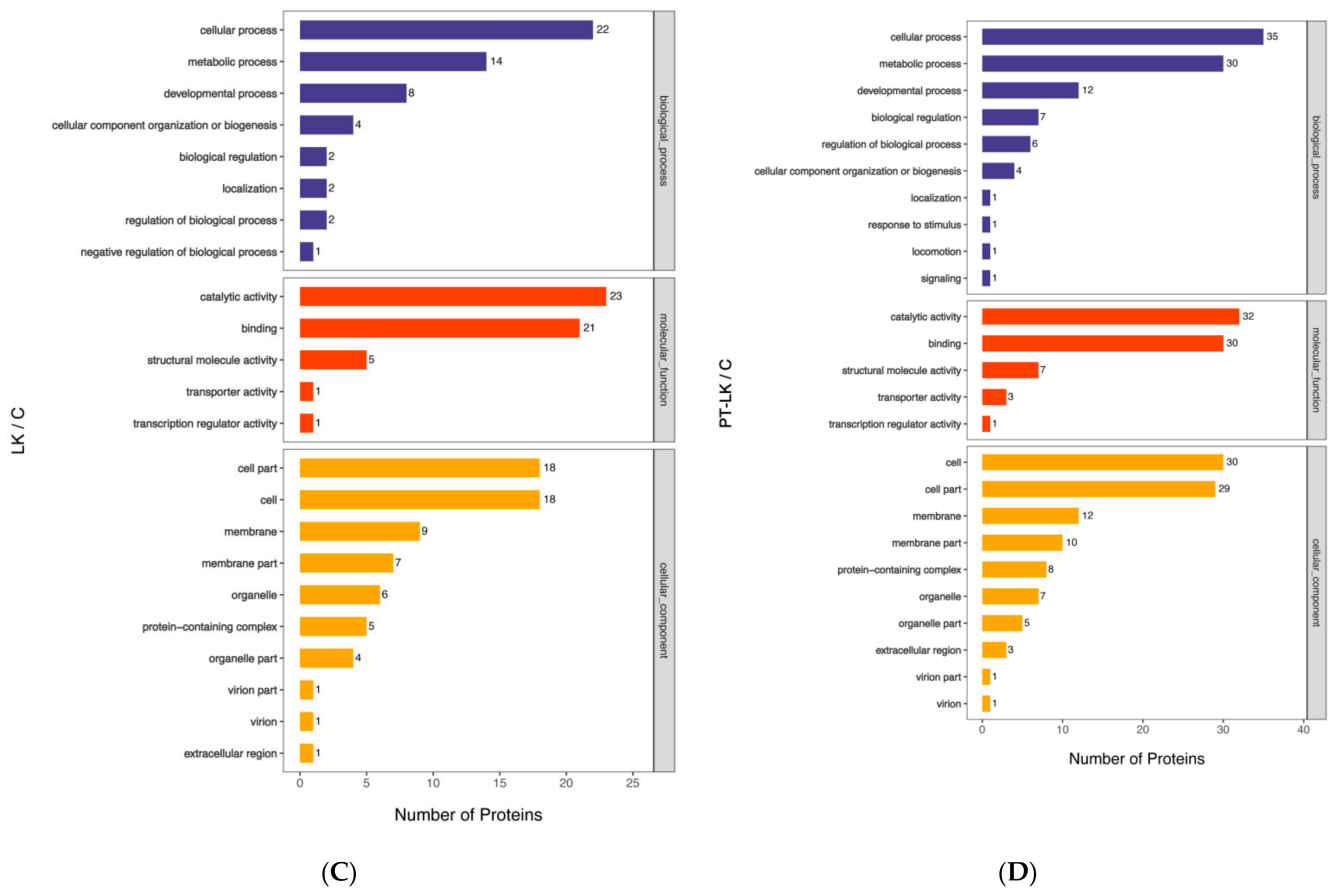
Functional categorization based on GO level 2 analysis of significantly differentially abundant *B. subtilis* spore proteins after different treatments: (**A**) P/C, (**B**) T/C, (**C**) LK/C, and (**D**) TP-LK/C.

The results of the GO level 2 analysis are presented in Figure 7. Within the 181 DEPs in the P/C group, 270 proteins played a role in 15 different biological processes, 218 proteins had seven distinct molecular functions, and 236 proteins were related to 11 cellular components. In contrast with the control group, 10 DEPs in the T group comprised 11 proteins that participated in five biological processes, while 12 proteins had two specific molecular functions, and 12 proteins were related to five different cellular components. There were 49 DEPs in the LK/C comparison, with 55 proteins in eight biological processes, 56 proteins with five molecular functions, and 70 proteins with 10 cellular components. It was found that 78 DEPs in the TP-LK group comprised 198 proteins that had 10 biological processes, 73 proteins that had under five molecular functions, and 106 proteins that had 10 cellular components. Biological process analysis revealed that the cellular process mainly included proteins in all four comparisons. Metabolic process proteins achieved second position among P/C, LK/C, and PT-LK/C comparisons, while cellular component organization or biogenesis proteins were ranked second in T/C.

Molecular function analysis indicated that catalytic activity and binding described the majority of proteins in all four comparisons. Cellular component analysis revealed that the cell, cell part, membrane, membrane part, and organelle were involved the majority of proteins in all four comparisons. It is worth noting that even PT-LK had an effect on *B. subtilis* spore inactivation, but high pressure with 200 MPa targeted more proteins, which might correspond to the effect of moderate temperature on bacteria spores.

### 3.5. KEGG Pathway Analysis of DEPs after Different Treatments

Analysis of the KEGG pathway was performed to explain the biological processes related to the DEPs induced by P, T, LK, and PT-LK treatment.

#### 3.5.1. Roles of DEPs in P/C Regulation

**Figure 8:**
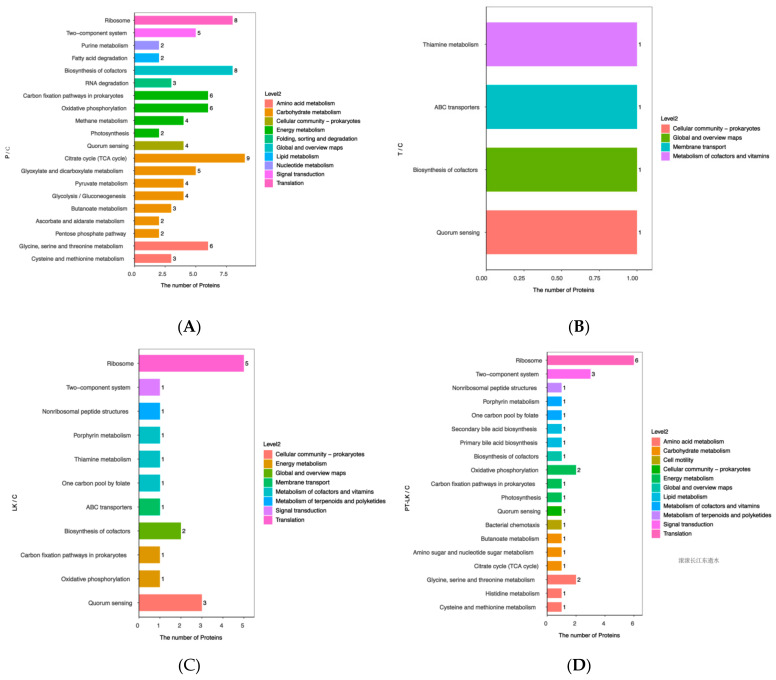
Distribution of DEPs in *B. subtilis* spores in KEGG level 2 in response to different treatments: (**A**) P/C, (**B**) T/C, (**C**) LK/C, and (**D**) TP-LK/C.

It can be seen from the data in Figure 8 that DEPs in P were mainly involved in metabolism and translation pathways as compared to the control. Additionally, proteins were mainly related to the citrate cycle (TCA cycle), ribosome, oxidative phosphorylation, biosynthesis of cofactors, carbon fixation pathways in prokaryotes, glyoxylate and dicarboxylate metabolism, the two-component system, glycine, serine, and threonine metabolism pathways. As shown in Table 2, the TCA cycle pathway included 2-oxoglutarate dehydrogenase E1 component (P23129), aconitate/2-methylaconitate hydratase (P09339), malate dehydrogenase (P49814), pyruvate carboxylase (Q9KWU4), dihydrolipoyllysine-residue succinyltransferase component of 2-oxoglutarate dehydrogenase complex (A0A6A8FKK9), phosphoenolpyruvate carboxykinase (ATP) (A0A7U5BTG7), succinate-CoA ligase (ADP-forming) subunit alpha (A0A5F2KMI5), succinate dehydrogenase flavoprotein subunit (A0A3N6CX89), and succinate-CoA ligase (ADP-forming) subunit beta (A0A3A5I5U1). The ribosome pathway contained 30S ribosomal protein S13 (P20282), 50S ribosomal protein L5 (P12877), 30S ribosomal protein S1 homolog (P38494), 30S ribosomal protein (S2P21464), 50S ribosomal protein L13 (M4KMS5), 50S ribosomal protein L29 (D4G3L1), 30S ribosomal protein S4 (A0A4R6HVR7), and 30S ribosomal protein S14 (A0A5D4N259).

Among the upregulated proteins, in the TCA cycle pathway, the 2-oxoglutarate dehydrogenase E1 component (P23129), the E1 component of the 2-oxoglutarate dehydrogenase (OGDH) complex catalyzing the decarboxylation of 2-oxoglutarate, is the first phase in the conversion of 2-oxoglutarate to succinyl-CoA and CO. Aconitate/2-methylaconitate hydratase (P09339) is capable of catalyzing the reversible isomerization of citrate to isocitrate via *cis*-aconitate and the rehydration of 2-methyl-cis-aconitate to produce 2-methylisocitrate, which is an essential step in the tricarboxylic and methylcitric acid cycle. During spore formation, the apo form of AcnA also acts as a regulatory protein to adjust citrate concentration, and aconitase combines with the gerE transcript that stabilizes its translation and added the accumulation of gerE protein in late-stage formation. Pyruvate carboxylase (Q9KWU4), which is in charge of catalyzing a two-step reaction, including the ATP-dependent carboxylation and the carboxyl group’s transfer to pyruvate, performs an anaplerotic function in *B. subtilis* because it is necessary for glucose growth rather than spore production. In addition, succinate–CoA ligase (ADP-forming) subunit alpha (A0A5F2KMI5) and beta (A0A3A5I5U1) succinyl–CoA synthetase function in the TCA, coupling the hydrolysis of succinyl–CoA with the synthesis of either ATP or GTP.

In the ribosome pathway, the 30S ribosomal protein S13 (P20282), located at the top of the 30S subunit, is in contact with multiple helices of the 16S rRNA. The 70S ribosome contacts tRNAs in the A and P sites, as well as 23S rRNA (bridge B1a) and protein L5 of the 50S subunit (bridge B1b). Both bridges are related to the movement of the subunit. The 50S ribosomal protein L5 (P12877), as a 5S RNA binding protein, may mediate the 5S RNA attachment to the large ribosomal subunit and is involved in the formation of the central protrusion. The 70S ribosome makes contact with protein S13 of the 30S subunit (bridge B1b). Thus, the bridge is associated with the movement of the subunits. Moreover, it also contacts the P-site tRNA and helps stabilize the position of the ribosome-bound tRNA, which is attributed to the 5S rRNA and its correlated proteins. The 30S ribosomal protein S1 homolog (P38494) plays a role in sporulation. In addition, corresponding protein S4 (A0A4R6HVR7) and protein S14 (A0A5D4N259) are both critical 16S rRNA-binding proteins; the former affects the accuracy of translation when bonded to S5 and S12, while the latter determines the A site of the conformation. The 50S ribosomal protein L13 (M4KMS5) is an essential early assembly protein of the 50S ribosomal subunit but not a 16S rRNA-binding protein.

#### 3.5.2. Roles of DEPs in T/C Regulation

In contrast with the control group, there were only four DEPs with *p* > 0.05 in the treated group at 80 °C, including thiamine metabolism, ABC transporters, biosynthesis of cofactors, and quorum sensing (Figure 8), which means that moderate treatment resulted in no significant differences. In the germination experiment, spores were usually pretreated at 80 °C, ensuring the release of DPA in the spore with the treatment of germinants, such as V-ala, V-val, and AGFK, which subsequently activate the pathway of spore germination. Temperature is considered to be the key factor for spore germination and inactivation. When the treatment condition was 80 °C, the temperature-sensing proteins located in the spore would been activated through the transmission of the temperature signal by the receptor protein on the membrane of spores, which could also be related to the abnormal expression of ABC transporters.

#### 3.5.3. Roles of DEPs in LK/C Regulation

Compared with the control (Figure 8), the DEPs in the LK group were mainly involved in genetic information processing, the cellular process, and metabolism. Furthermore, proteins were mainly related to biosome, quorum sensing, biosynthesis of cofactors, one-carbon pool by folate, thiamine metabolism, porphyrin metabolism, non-ribosomal peptide structures, oxidative phosphorylation, carbon fixation pathways in prokaryotes, the two-component system, and ABC transporter pathways. As shown in Table 3, the ribosome pathway mainly covered 30S ribosomal protein S13 (P20282), 30S ribosomal protein S2 (P21464), 30S ribosomal protein S7 (A0A7Z9E609), 30S ribosomal protein S4 (A0A4R6HVR7), and 50S ribosomal protein (A0A1A0G613). The quorum sensing pathway involved ComQ (Q38HY3) and ABC transporter ATP-binding protein (A0A5D4N383). The biosynthesis of cofactors pathway mainly included coproporphyrin III ferrochelatase (A0A7U5AV14) and glycine oxidase ThiO (A0A164STP7).

#### 3.5.4. Roles of DEPs in PT-LK/C Regulation

From the data in Figure 8, a comparison of the TP-LK and C groups revealed that DEPs were mainly involved in genetic information processing, metabolism, cellular process, and environmental information processing. In addition, proteins mainly belonging to pathways were related to ribosome, the two-component system, oxidative phosphorylation, secondary bile acid biosynthesis, bacterial chemotaxis, photosynthesis, one-carbon pool by folate, porphyrin metabolism, butanoate metabolism, non-ribosomal peptide structures, histidine metabolism, carbon fixation pathways in prokaryotes, amino sugar and nucleotide sugar metabolism, cysteine and methionine metabolism, TCA, quorum sensing, biosynthesis of cofactors, and metabolism of glycine, serine, and threonine.

As shown in Table 4, the ribosome pathway included 30S ribosomal protein S13 (P20282), 50S ribosomal protein L2 (P42919), 30S ribosomal protein S2 (P21464), 30S ribosomal protein S7 (A0A7Z9E609), 30S ribosomal protein S4 (A0A4R6HVR7), and 50S ribosomal protein L21 (A0A1A0G613).

The two-component system pathway included signal transduction histidine protein kinase/phosphatase DegS (P13799), teichoic acid d-alanyltransferase (A0A3A5HYZ2), and chemotaxis protein CheA (A0A8B5NH90). The 50S ribosomal protein L2 (P42919) is suggested to have peptidyltransferase activity, and 50S ribosomal protein L21 (A0A1A0G613), as a member of the two-component regulatory system DegS/DegU, plays an essential role in the transition growth phase. It makes contact with 16S rRNA in the 70S ribosome. The 50S ribosomal protein L21 (A0A1A0G613) binds to 23S rRNA in the presence of protein L20. It regulates the expression of several cellular processes, including the generation of degradative enzymes such as neutral and alkaline proteases, flagellum development, and biofilm formation. It functions as a protein kinase that auto-phosphorylates and transfers the phosphate to DegU and a protein phosphatase that dephosphorylates phospho-DegU. Teichoic acid d-alanyl transferase (A0A3A5HYZ2) may be engaged in transporting activated d-alanine via the membrane.

The oxidative phosphorylation pathway includes ATP synthase subunit c (P37815) and cytochrome c oxidase subunit 2 (A0A0G2YRU4). The ATP synthase subunit c (P37815) consists of F(1) and F(0) domains, and ATP synthase is generated under the existence of a proton or sodium gradient. The F-type ATPase comprises two structural regions, F(1) containing the extra-membrane catalytic core and F(0) containing the membrane proton channel, linked together by a central stalk and a peripheral stalk. Cytochrome c oxidase subunit 2 (A0A0G2YRU4) and subunits I and II form the functional core of the enzyme complex, which is a binuclear center derived from the formation of electron transfer from cytochrome c.

### 3.6. Subcellular Localization of DEPs after PT-LK Treatment

**Figure 9:**
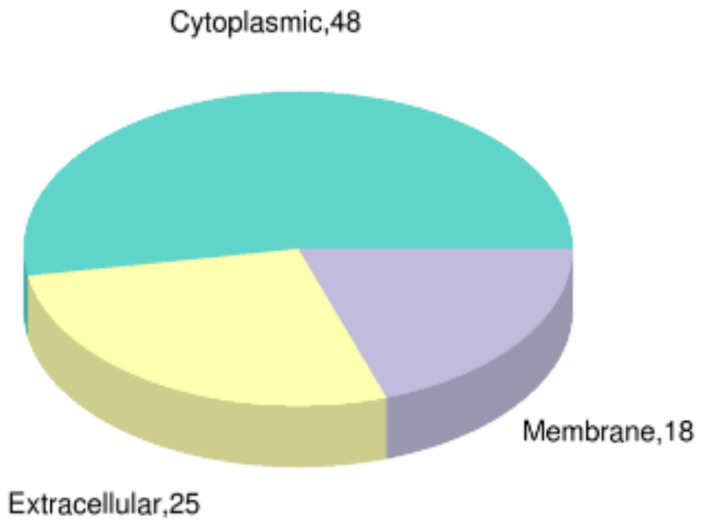
Subcellular localization of DEPs in *B. subtilis* spores after PT-LK treatment.

Subcellular organelles such as the mitochondria and endoplasmic network are micro-organs with a certain morphology and function in the cytoplasm, which is an important place for the various functions of proteins. Different subcellular organelles tend to exercise different cellular functions; thus, analyzing the subcellular localization of proteins helps us to further explore the functions that proteins have in cells. The cytoplasm is where cellular life activities take place, such as protein synthesis and respiration. The subcellular localization of *B. subtilis* spores after PT-LK treatment is presented in Figure 9, with the cytoplasm accounting for 52.5% of the total distribution with 48 DEPs. Membranes with 18 DEPs and extracellular space with 25 DEPs covered the remaining regions of subcellular localization. The subcellular localization suggested that proteins in the cytoplasm, membrane, and extracellular space had corresponding stress responses after PT-LK treatment. This is consistent with our previous research, where PT-LK treatment could induce cortex hydrolysis and damage spore structure, especially in the IM.

## 4. Discussion

High-pressure thermal sterilization (HPTS) is a promising technology built on HPP with an application of moderate pressure and a lower thermal load to sterilize food while retaining food quality. An increase in the LK concentration to 10^2^ μg/mL combined with HPTS highly accelerated the spore inactivation rate for all tests. Following this synergistic effect, cortex hydrolysis and IM damage were observed from both fluorescence microscope images and FMC analysis. It can be seen that the PT-LK synergistic effect caused the spore structure to change, which in turn led to the inactivation of all spores by 8.16 log. This result is consistent with previous reports [30]. In addition to maintaining a higher quality of food products, shelf-life extension results in less food loss. However, the moderate pressure and lower cost of RPM synthesis within 15 min indicate lower energy consumption. So far, the wear and tear on HPP equipment of the vessel and seals combined with temperature is a problem in this method of sterilization that needs to be solved. Additionally, this method has not been tested in any specific food systems; hence, future research must focus on this.

Fluorescence microscope images and FCM analysis showed that spores with different treatments displayed different fluorescence intensities. We found that, in the same treatment time of 15 min, all spores with PT-LK treatment germinated and the membrane permeability increased, although the individual treatments with a pressure of 200 MPa, temperature of 80 °C, or 10^2^ μg/mL LK could kill the same spore with a changed membrane structure. These results suggested that the treatment of PT-LK displayed a synergistic sterilization effect and could kill 8.16 log spores more effectively, potentially due to the damage of the membrane structure. Previous reports indicated that the complex structure of spores with multiple layers can play a specific role in their resistance. From the inside out, the various spore layers include the core, IM, germ cell wall, cortex, outer membrane (OM), coat, and exosporium [30]. However, only some species, such as the pathogenic organisms *B. cereus* and *B. anthracis*, have an exosporium. In *B. subtilis*, the outermost spore structure is a coat that acts as a permeability barrier by limiting access of enzymes and some other large molecules that attempt to steal potential sensitive targets inside of the spore [31]. Thus, the spore coat, with its large number of proteins, is highly resistant and can protect spores against degradative enzymes [32]. In addition to the spore coat, the cortex and germ cell wall underlying the OM are essential for spore viability, especially for the cortex, which can prevent the expansion of spore core to maintain spore dormancy [33]. In view of its low mobility [34], low permeability, and high viscosity [35], the IM plays an important role in spore resistance and germination. However, all of the IM’s properties are lost when spores complete germination [30]. Last but not least, the center of spore is the core, which has a very low water content (which contributes to wet heat resistance of spore), a high level of DPA (which is important in spore resistance and dormancy), and numerous proteins, which protect the spore DNA against harsh environments [36]. In the study, it was demonstrated that the PT-LK synergistic effect caused the spore structure to change, which in turn led to the inactivation of all spores by 8.16 log.

The proteomic response of *B. subtilis* spores under TP-LK treatment indicated that most proteins were involved in genetic information processing, metabolism, the cellular process, and environmental information processing. Moreover, proteins mainly belonged to pathways related to ribosomes, the two-component system, oxidative phosphorylation, secondary bile acid biosynthesis, bacterial chemotaxis, photosynthesis, one-carbon pool by folate, porphyrin metabolism, butanoate metabolism, non-ribosomal peptide structures, histidine metabolism, carbon fixation pathways in prokaryotes, amino sugar and nucleotide sugar metabolism, cysteine and methionine metabolism, the TCA cycle, quorum sensing, biosynthesis of cofactors, and metabolism of glycine, serine, and threonine. More importantly, the synthesis of ATP was a very important factor for restricting spore germination, whereas PT-LK significantly influenced the related pathways.

## 5. Conclusions

Dormant spores from *Bacillus* and *Clostridium* species are extremely resistant to treatments that inactivate growing bacteria, and these bacteria can cause some severe human diseases. Therefore, spore inactivation has attracted more and more attention. High hydrostatic pressure technology is a nonthermal sterilization method, which can kill spores through combination with other treatment conditions including heat and inhibition. In this study, the effect of the combination of PATP, a promising technology, with the application of moderate pressure, low temperature, and antimicrobial peptide LK to inactivate *B. subtilis* spores, was studied. The combined use of HPTS and LK had a powerful synergistic effect on the inactivation of spores and generated more damage to the spore structure, including cortex hydrolysis and IM destruction. Furthermore, the proteomic response of *B. subtilis* spores under PT-LK treatment indicated that most proteins were involved in genetic information processing, metabolism, the cellular process, and environmental information processing. Our results provide evidence that antimicrobial peptide LK could kill about 8 log *B. subtilis* spores under conditions of 200 MPa at 80 °C for 15 min, the effect of which depended on the treatment time and the concentration of LK. These results demonstrated that PT-LK treatment has great potential as an effective and reliable sterilization technique for dormant spores.

## Figures and Tables

**Table 1:** Percentage of inactivated spores with different percentage gating in FCM density plots.

Treatment	% Gated			
	UL	UR	LL	LR
Untreated	12.24 ± 0.54 ^f^	17.69 ± 1.11 ^e^	61.83 ± 1.93 ^c^	8.23 ± 1.1 ^fg^
200 MPa 15 min	0.06 ± 0.04 ^i^	76.89 ± 1.42 ^b^	2.55 ± 0.47 ^hi^	20.51 ± 1.48 ^e^
80 °C 15 min	0.41 ± 0.31 ^i^	50.06 ± 2.56 ^d^	3.02 ± 1.65 ^hi^	46.52 ± 3.96 ^d^
10^2^ μg/mL LK 25 °C 15 min	0.06 ± 0.06 ^i^	46.59 ± 3.68 ^d^	3.82 ± 0.46 ^ghi^	49.53 ± 3.57 ^d^
200 MPa 80 °C 10^2^ μg/mL LK 15 min	0.35 ± 0.11 ^i^	82.18 ± 1.44 ^a^	6.28 ± 1.07 ^gh^	11.2 ± 2.28 ^f^

Values are the mean of triplicate measurements ± standard deviation; values with different lowercase letters represent a significant difference according to ANOVA test (*p* < 0.05).

**Table 2:** The respective DEPs in *B. subtilis* spores against P treatment.

Protein Description	Test Sequences	*p*-Value	Rich Factor
Citrate cycle (TCA cycle)			
2-Oxoglutarate dehydrogenase E1 component	P23129	0.00	0.18
Aconitate/2-methylaconitate hydratase	P09339	0.00	0.18
Malate dehydrogenase	P49814	0.00	0.18
Pyruvate carboxylase	Q9KWU4	0.00	0.18
Dihydrolipoyllysine-residue succinyltransferase component of 2-oxoglutarate dehydrogenase complex	A0A6A8FKK9	0.00	0.18
Phosphoenolpyruvate carboxykinase (ATP)	A0A7U5BTG7	0.00	0.18
Succinate–CoA ligase [ADP-forming] subunit alpha	A0A5F2KMI5	0.00	0.18
Succinate dehydrogenase flavoprotein subunit	A0A3N6CX89	0.00	0.18
Succinate–CoA ligase [ADP-forming] subunit beta	A0A3A5I5U1	0.00	0.18
Ribosome			
30S ribosomal protein S13	P20282	0.03	0.13
50S ribosomal protein L5	P12877	0.03	0.13
30S ribosomal protein S1 homolog	P38494	0.03	0.13
30S ribosomal protein S2	P21464	0.03	0.13
50S ribosomal protein L13	M4KMS5	0.03	0.13
50S ribosomal protein L29	D4G3L1	0.03	0.13
30S ribosomal protein S4	A0A4R6HVR7	0.03	0.13
30S ribosomal protein S14	A0A5D4N259	0.03	0.13
Oxidative phosphorylation			
Quinol oxidase subunit 1	P34956	0.02	0.15
ATP synthase subunit c	P37815	0.02	0.15
ATP synthase subunit alpha	A0A0D1KS60	0.02	0.15
Cytochrome c oxidase subunit IVB	A0A5F2KHE8	0.02	0.15
Succinate dehydrogenase flavoprotein subunit	A0A3N6CX89	0.02	0.15
Cytochrome c oxidase subunit 2	A0A0G2YRU4	0.02	0.15
Glycine, serine, and threonine metabolism			
Betaine aldehyde dehydrogenase	P71016	0.04	0.14
Aminomethyltransferase	P54378	0.04	0.14
l-Serine ammonia-lyase, iron-sulfur-dependent subunit beta	A0A8A7MTS3	0.04	0.14
Hydroxymethyltransferase	A0A857HNR6	0.04	0.14
Glycine dehydrogenase (Decarboxylating) subunit 1	A0A857HK36	0.04	0.14
NAD-dependent alcohol dehydrogenase	A0A8B5NR48	0.04	0.14
Carbon fixation pathways in prokaryotes			
Aconitate/2-methylaconitate hydratase	P09339	0.04	0.14
Malate dehydrogenase	P49814	0.04	0.14
Pyruvate carboxylase	Q9KWU4	0.04	0.14
Succinate–CoA ligase (ADP-forming) subunit alpha	A0A5F2KMI5	0.04	0.14
Succinate dehydrogenase flavoprotein subunit	A0A3N6CX89	0.04	0.14
Succinate–CoA ligase (ADP-forming) subunit beta	A0A3A5I5U1	0.04	0.14

**Table 3:** The respective DEPs in *B. subtilis* spores against LK treatment.

Protein Description	Test Sequences	*p*-Value	Rich Factor
Ribosome			
30S ribosomal protein S13	P20282	0.00	0.078
50S ribosomal protein L21	A0A1A0G613	0.00	0.078
30S ribosomal protein S2	P21464	0.00	0.078
30S ribosomal protein S7	A0A7Z9E609	0.00	0.078
30S ribosomal protein S4	A0A4R6HVR7	0.00	0.078

**Table 4:** The respective DEPs in *B. subtilis* spores after PT-LK treatment.

Protein Description	Test Sequences	*p*-Value	Rich Factor
Ribosome			
30S ribosomal protein S13	P20282	0.00	0.09
50S ribosomal protein L2	P42919	0.00	0.09
30S ribosomal protein S2	P21464	0.00	0.09
30S ribosomal protein S7	A0A7Z9E609	0.00	0.09
30S ribosomal protein S4	A0A4R6HVR7	0.00	0.09

## Data Availability

The data presented in this study are available on request from the corresponding author.
